# Introduction: Perspectives on Democracy

**DOI:** 10.1007/s11615-020-00252-4

**Published:** 2020-05-14

**Authors:** Ferdinand Müller-Rommel, Brigitte Geißel

**Affiliations:** 1 Leuphana Universität Lüneburg, Lüneburg, Germany; 2 Frankfurt am Main, Germany

**Keywords:** Democratic theory, Democratic innovation, Participatory reforms, New populism, Social media, Globalization, Deliberation, Direct democracy, Participatory systems, Demokratietheorie, Demokratische Innovation, Partizipatorische Reformen, Neuer Populismus, Soziale Medien, Globalisierung, Demokratische Innovationen, Deliberation, Direktdemokratie, Partizipative Systeme

## Abstract

This article explores diverse views on both the current challenges and limits as well as the reforms and innovations of existing democracies at the beginning of the twenty-first century. First, it argues that socioeconomic inequality, new populism, new forms of communication, and globalization have stimulated a renewal of interest in analyzing the “frontiers of democracy.” Democracies have reacted with different innovations and reforms in order to meet these challenges. The authors trace the phases of respective research from studies on singular, standalone instances to normative as well as empirical work on participatory (direct democratic and deliberative) systems. Finally, they advocate for combining the conceptual approach of defining democracy by the fulfillment of democratic values with rigorous empirical evaluation of the contributions (old and new) that institutions and procedures provide in order to fulfill these values and meet the mentioned challenges.

## Abstract (de)

Dieser Artikel untersucht unterschiedliche Ansichten über die aktuellen Herausforderungen und Grenzen sowie auch über Reformen und Innovationen der bestehenden Demokratien zu Beginn des 21. Jahrhunderts. Er verdeutlicht, dass die sozioökonomische Ungleichheit, der neue Populismus, die neuen Kommunikationsformen und die Globalisierung zu einem neuen wissenschaftlichen Diskurs über die „Grenzen der Demokratie“ geführt haben. Demokratien haben mit unterschiedlichen Innovationen und Reformen auf diese Herausforderungen reagiert. Die Autoren zeichnen die Phasen der Demokratische-Innovationen-Forschung nach – von der Fokussierung auf einzelne Innovationen bis zu normativen und empirischen Studien zu partizipativen (direktdemokratischen und deliberativen) Systemen. Der Beitrag endet mit einem Plädoyer für zukünftige politikwissenschaftliche Forschung. Notwendig ist die Kombination eines konzeptuellen Zugangs, bei dem Demokratie anhand der Verwirklichung demokratischer Werte definiert wird, mit gründlicher empirischer Evaluation der Beiträge (neuer und alter) Institutionen und Verfahren zur Erfüllung dieser Normen sowie zur Bewältigung der genannten Herausforderungen.

## Preface

This special issue examines recent challenges to democracy as well as reforms and innovations for the future of democratic regimes across the world. It is based on working papers that were presented at the National Congress of the German Political Science Association (DVPW/GPSA) in Frankfurt (autumn 2018). All articles in this special issue are therefore closely linked to the main theme of the congress, titled “Frontiers of Democracy.” The congress aimed to introduce diverse views on both the current challenges and limits as well as the reforms and innovations of existing democracies at the beginning of the twenty-first century.

This special issue brings together a group of distinguished scholars from several subdisciplines of political science, who look at these issues from different angles and provide selected narratives with thorough conceptual considerations as well as empirical examinations. This issue thereby intends to reflect on the present and the future of democracy from a panorama lens in order to understand the broad range of crucial challenges and innovative developments within liberal democracy. Obviously, the following articles will not answer all questions raised in the literature over the past decades, but they might (hopefully) reopen a crucial discussion about the development and the functioning of democratic regimes from a cross-national perspective.

## Challenges to Democracy

In democratic theory, most scholars agree with the definition that democracy means inclusive, collective (or at least collectively accepted) will formation and decision making, aiming at political responsiveness—in the sense of effective transformation of citizens’ preferences into policies and outcomes—while ensuring political rights and liberties via constraints of the will of the people (Warren 2017). Yet this abstract definition gives no clear indication about how people are meant to rule. Therefore, the normative idea of democracy must be related to the concrete functioning of political institutions in a nation state, as expressed in the rule of law. As Juan Linz (1997, p. 120) has rightly put it, “No state (…) no democracy.” What this statement exactly means in practice has been examined in many different and sometimes overlapping conceptual definitions. By the end of the1990s, the concept of democracy—whether, for example, liberal, transnational, associative, social, procedural, substantive, deliberative, global, emancipative, electoral, or inclusive—was on the agenda of more than 500 different scholars in the field of empirical research and normative theory (Collier and Levitsky 1997).

The majority of these studies accepted liberal democracy as the only game in town. With the European Union as a poster child of supranational cooperation, even a new, postnational democratic order seemed possible. The future looked bright, with a peaceful world in which national and international conflicts could be solved by democratic means of law and negotiation. However, recent events and developments have shown that the present and the future of democracy do not look as bright. At the beginning of the twenty-first century, liberal democracies are faced with at least four clusters of challenges:

First, the *socioeconomic patterns* of many liberal democracies have changed substantially. For one, population growth in liberal democracies is declining in spite of heavy immigration of citizens from other regime types. Furthermore, the number of younger cohorts is decreasing, while the sizes of older cohorts are increasing. Regarding the occupational structure, both the overall numbers of unemployed citizens and the proportion of female participation in the labor force are also increasing. Moreover, industrial employment is in decline, whereas the public and private sectors of the economy are growing. In light of these changes, the rise of political and socioeconomic inequality has dramatically challenged contemporary democracies. Not only in Germany but all over the democratic world, the rich are getting richer and the poor are getting poorer. Marginalization of the “have-nots” has led to ideological divergence from the democratic mainstream as well as to alienation from the established parties and other political institutions.

Second, as a consequence of the socioeconomic inequality and the growing of new social cleavages, various liberal democracies are challenged by *new forms of populism* that have emerged on the right and the left sides of the ideological spectrum. On the one hand, right-wing populism has existed for more than 20 years in all major liberal democracies as a reaction against neoliberal economies, immigration policies, and international (global) political actors. It defends a right-wing national cultural identity and the revitalization of the nation-state. On the other hand, conservative left-wing populists also favor the nation-state by defending a national welfare policy as well as domestic production sites. Both populist movements are organized in political parties. Right-wing populist parties exist in nearly all European democracies, while populist parties of the left exist particularly in Mediterranean countries (e.g., MoVimento 5 Stelle in Italy, Syriza in Greece, and Podemos in Spain). Their electoral success has fundamentally challenged the party systems as well as party governments in several parliamentary democracies at different times (Kriesi and Pappas, 2015; Kriesi 2014, p. 369).

The political impact of these populist parties on liberal democracies can be twofold: On the one hand, new populism may lead to a “popular democracy” without a competing party system. In this scenario, neopopulist actors point out the political failures of established parties, in particular their decreasing function as mediators between citizens and their governments within the electoral channel. As a consequence, voter support for the established parties is radically decreasing and leading to new forms of democratic governance that operate without political parties. Thus, populism may lead to “partyless democracy” that replaces “constitutional democracy” (Mair 2002, p. 89).

On the other hand, new populism can be viewed as a productive force that may profoundly realign existing party systems. For instance, left-wing and right-wing populist parties may affect the political issues and the tone of political life by bringing controversial matters in front of the public. They may, therefore, serve as political vehicles for those voters whose grievances have been ignored by the larger established parties. By making themselves the spokespersons of the discontented, populist parties have mobilized many voters and thereby have promoted a change of party loyalties, which in turn has paved the way for electoral volatility within parliamentary party systems. This realignment supports a democratic process of party competition for votes and governmental power (Kriesi 2014; Müller-Rommel 2016).

Third, liberal democracies have experienced a changing structure of *political communication*. The digital revolution, and particularly the internet, social media, and smartphone applications, has led to major technological innovations providing citizens and also parties, governments, and other political institutions with new forms of communication (Wewer 2014). Yet the effect of the digital world on the quality of democracy is still unclear. On the one hand, it is well known that contemporary democracies depend heavily on the infrastructure of the new communication systems. Modern campaigning, for instance, is not conceivable anymore without the use of social media (e.g., Twitter, Facebook, YouTube) that allows for a closer link between voters and candidates. In this sense, digitalization provides more opportunities for participating and thereby initiates new forms of politicizations. On the other hand, digital media and digital communication can be disruptive to the quality of democracy. For instance, during the crisis of the coronavirus, several democratic states have suggested tracking the individual activities of citizens in order to control the spread of the virus. While this tracking obviously helps to collect huge amounts of geolocation data to identify infected people, it also risks crossing the line of data privacy. As long as the state officially asks citizens to use the data for health services, the door remains open for democratic accountability. Yet if the acceleration of technology is increasingly operated by private social media platforms, the information environment may be manipulated, and fake news may spread quickly on the various platforms. Against this background, a new public debate about the interrelations between political communication, technological progress, and the protection of basic democratic rights and values is overdue.

Finally, *globalization* challenges liberal democracies in at least three ways. First, democratic decisions made in one country might affect the lives of citizens in neighboring countries. This is nothing new and has always occurred in the history of nation-states. However, in an interconnected world with open borders, an open labor market, and transnational educational programs, sovereign political decisions of one state (e.g., on environmental, economic, or financial policies) might have a larger effect on citizens living in another state. Citizens of one country may, for instance, feel that their interests are not represented in the decision making of the authorities in their neighbor countries. In light of an increasing social and economic inequality that is currently observed across liberal democracies, it can be expected that these transborder effects of decision making are likely to expand and thereby might create larger problems in transnational political decision making (Kriesi 2013, p. 4).

Second, in a world of global interdependencies and multilevel governance structures, the issue of political representation becomes crucial. The activities of supranational political actors, such as the International Monetary Fund, the World Bank, the United Nations, and the World Trade Organization, cannot be directly controlled by any national state. However, their decisions increasingly influence national social and economic policies and thereby impinge on the well-being of many citizens. Currently, party governments in liberal democracies need to represent the political interests of their national citizens in order to stay in power or gain power. At the same time, they need to fulfill their obligations toward the requirements of international organizations, which often contravene the interests of the voters in single countries. This growing and potentially unbridgeable gap between “responsiveness and responsibility of party government” has a negative impact on representative democracy (Mair 2009, p. 16 f). In this sense, globalization may lead to a depoliticization of citizens and national political parties as well as to “mixed governance structures” in which nongovernmental organizations and national governments will secure “intergovernmental democracy.”

Third, liberal democracies might simultaneously be threatened by external shocks on the cosmopolitan level. As this introductory note is being written, the global world is being challenged by the COVID-19 pandemic. This ongoing crisis will have unpredictable consequences for the development of civil liberties and liberal values in established democracies. Some governments have used this health crisis to impose dramatic measures at the expense of fundamental democratic rights, such as placing limitations on citizens’ free movements as well as on the freedom to associate and assemble. But in most countries these measures were implemented by robust democratic institutions and imposed with clear expiration dates. In these mature democracies, the political order will most likely return to normal after the pandemic crisis. However, it remains unclear whether some of the extraordinary restrictions in people’s lives will stay in place, if only as preemptive measures for the future. The antiterror laws that were implemented in several European countries some decades ago show, for instance, that some of the rigid policies will remain part of liberal democracies even after the threat subsides.

In more fragile democracies, the challenges of COVID-19 are more substantive. Several “imperfect democracies” with less resilient economies, higher levels of inequality, and weak social security systems were already forced by democratic backsliding before the crisis. In these countries, the proclamation of emergency measures has been taken up as an excuse to tighten the power of politicians with authoritarian leadership styles. One prominent case is Hungary, where Prime Minister Orbán used his party majority in parliament to allow him to rule the country by decree indefinitely without having to be accountable to anyone. These and other restrictions of civil and political rights may foster already embattled democratic institutions in some new democracies.

These four major challenges in the areas of socioeconomic development, populism, communication technology, and globalization have stimulated a massive scientific renewal of interest in analyzing the “frontiers of democracy.” In a nutshell, academic scholars have—broadly speaking—predicted three different directions that liberal democracy may take: One direction emphasizes that democracy is “in crisis.” Scholars in this field proclaim the eroding of mature democracies. This alarming point is, for instance, made most prominently by Foa and Mounk, who talk about “the democratic disconnect” (2016) and the “signs of deconsolidation” (2017). One of their arguments is that the fading support for democracy is responsible for the populist turn among the electorates of most liberal democratic regimes. Other scholars speak of “democratic recession” (Levitsky and Way 2015), “democratic backsliding” (Bermeo 2016), and even the “death of democracy” (Keane 2009; Levitsky and Ziblatt 2018). From this point of view, democracy (in its current form) has clearly reached its limits.

The second view on contemporary democracies is more optimistic. Scholars including Merkel and Kneip (2018) argue on the basis of expert judgments, population surveys, and analysis of democratic institutions that there is no systematic empirical evidence for a crisis of democracy. Along similar lines, behavioral scientists such as Alexander and Welzel (2017), Inglehart (2016), and Norris and Inglehart (2019) claim that the level of democratic support is astonishingly high among all age cohorts in democratic societies. In a comparative and longitudinal analysis covering 116 years, Brunkert et al. (2019) found that public support for democracy is closely associated with emancipative values. The authors show that the increasing class polarization and marginalization of lower classes are the real source of the current decreasing dissatisfaction with political institutions and their decision making. Yet, at the same time, democracy is seen as a learning system in which liberal forces wake up once they are threatened by antisystem behavior. Precisely for that reason, the authors predict that liberal democratic regimes are born to survive.

The third stream of thought predicts that democracies become more “democratized.” The debates by Schmitter and Trechsel (2004) or Levine (2007) on the future of democracy as well the single contributions in *Democracy in Motion* (Nabatchi et al. 2012) are cases in point. In a nutshell, these conceptional approaches of democracy beyond elections are debated on a normative level and look for a “better” democracy, including the discussion of new forms of participatory institutions and procedures. Authors who follow this approach bid farewell to the narrow, “thin” concept of electoral democracy and advocate broad, “thick” concepts, including the evaluation of various forms of democratic reforms and innovations that are outlined in more detail in the following section (for a summary of the literature, see Geissel 2016, 2019).

## Reforms and Innovations for the Future of Democracy

Democratic systems have reacted in many different ways to these challenges. Among the most widespread reaction was the extension of participatory options, which does not address all challenges but is expected to solve some of them. Since the 1990s, most democracies have experienced vast transformation toward more participatory will formation and decision making by introducing direct democratic institutions at the national, regional, and local levels, as well as deliberative procedures such as the Danish consensus conferences, the deliberative constitutional procedures in Ireland, the Estonian Citizens’ Assembly, and G1000, a large-scale mini-public organized by Belgian civil society (overview for Europe: Geissel 2019).

In the academic world, these new forms of direct and deliberative democracy have been discussed widely (see the latest contributions in Escobar and Elstub 2019). Several hundred members of the European Consortium for Political Research Standing Group on Democratic Innovation have, for instance, discussed novel democratic concepts and conducted empirical research on a variety of reforms and innovations, such as economic effects of referendums, discourse quality within citizens’ assemblies, and the (dis)advantages of participatory budgeting within communities. Overall, they have concluded that democracy is more than elections and that elections do not make a democracy. Furthermore, they advocate introducing “thick” concepts of democracy to provide comprehensive options for citizens’ involvement.

Democratic reforms and innovations are defined in different ways, but in a general and widespread sense, they are understood as institutions and procedures designed to “democratize democracy.” Whereas reforms do not aim to challenge the representative system, innovations aim to provide new models beyond elections and representation. However, the boundaries between reforms and innovations are not carved in stone but are overlapping and fluid.

Research in this field of interest has developed—broadly speaking—in three phases: In the first phase, studies have primarily focused on insular, stand-alone participatory reforms and innovations, suggesting, describing, and evaluating single instances (Schmitter and Trechsel 2004). Well known in this context are studies on different types of referendums, recalls, or mini-publics (Michels 2011; Smith 2009). Although these publications are instructive and inspiring, they are limited in scope and method. Focusing on single instruments and procedures, they cannot provide a large picture.

In the second phase, the literature emphasized the need for conceptual perspectives beyond insular procedures. Although several authors had already stated that participatory and representative institutions and procedures do not compete but complement each other (e.g., Pogrebinischi 2013; Geissel and Newton 2012), it was the book by two political theorists, Parkinson and Mansbridge (2012), which significantly inspired the research community with the authorsʼ notion of “deliberative systems.” They argue that research has “to go beyond the study of individual institutions and processes to examine their interactions in the system as a whole” (2012, p. 2). Furthermore, different instruments and procedures “with multiple forms of communication among them” are recommended in order to generate a highly functional system (Mansbridge et al. 2012, p. 2). Warren (2017) presents another insightful contribution. He rejects the idea of defining democracy via a procedure and instead suggests a “problem-based approach.” He defines “normative requirements” of democracy, i.e., collective will formation and decision making, as well as inclusive empowerment, and argues that the combinations of different participatory and representative institutions and procedures would provide the best results. This normative literature is encouraging and insightful, but it can be described as (overly) enthusiastic, mainly emphasizing the positive aspects of participatory procedures and combinations. Potential drawbacks and trade-offs disappear behind the optimistic normative concepts.

In the current phase, scholars put increasingly more emphasis on empirical examinations of famous combinations, such as the participatory budgeting process in Porto Alegre, Brazil; Brazil’s National Public Policy Conferences; the British Columbia Citizens’ Assembly on Electoral Reform; and the Oregon Citizens’ Initiative Review. These studies show that on the one hand, combinations are often more effective than insular innovations. But on the other hand, they do not always provide the expected results and can even have detrimental effects such as (more) political frustration and disenchantment. This research is, however, still in its infancy, struggling with methodological challenges. There is still a long way to go from case-specific, problem-specific, and context-specific insights gained from a few examples to universally valid, generally accepted, and empirically sound findings, which are necessary to understand the dynamics and interactions between (new and old) institutions and procedures.

If we agree on the mission that political science should help us to govern ourselves (Mansbridge 2014, p. 8) and to make the world a better place (Fung and Wright 2003), the tasks for political science are clear-cut. Future research should be grounded in a normative and an empirical understanding of democracy: Democracy is to be understood normatively as inclusive, collective (or at least collectively accepted) will formation and decision making. Thus, a political system is to be considered democratic when democratic values are fulfilled—no matter which procedures are applied to fulfill these tasks, be it elections, direct democracy, or deliberation. This normative approach is to be combined with rigorous empirical studies on the impact of institutions and procedures of representative democracy as well as on changing values among citizens and their participatory behavior.

In summary, we argue that political institutions and procedures can be considered to be democratic only when they serve as a means to fulfill democratic values. Such an approach transcends the dichotomy of representation versus participation and deliberation versus aggregation in a way that conceptionally transcends the cleavages between established theories of representative, deliberative, and direct democratic democracy and allows for out-of-the-box thinking about democracy and the mentioned challenges.

## Exploring Diversity in Research on the “Frontiers of Democracy”

The single articles in this special issue implicitly refer to one of the major challenges to contemporary democracies or to reforms and innovation processes for the future of democracies. In doing so, they describe and discuss a variety of new theoretical and empirical findings that significantly contribute to the debate about the “frontiers of democracy.”

In the second article of this volume, *Hanspeter Kriesi* discusses the controversial question of whether or not democracy is “in crisis.” He provides empirical evidence that there is reason to be concerned about the development of liberal democracy but no reason to dramatize. His assessment is based on four perspectives: long-term trends revealed by quality of democracy measurements, citizens’ attitudes toward democratic principles and democracy in their own countries, the rise of populist challengers, and the elites’ perspective. Based on a variety of data sets, the author claims that despite widespread political dissatisfaction, liberal democracies will remain stable. This holds true even with populists in power because institutional constraints, partisan constraints, international market constraints, and constraints imposed by the citizens will hinder wider electoral success of right-wing populists. Thus, the existing hypothesis of contemporary democracies being in crisis is seen as largely exaggerated.

The third contribution examines the political impact of new populism on voting behavior in liberal democracies. *Hanna Schwander, Dominic Gohler, and Armin Schäfer* confirm the hypothesis that neither left-wing nor right-wing populist parties’ success alters the relationship between economic inequality and voter turnout. Analyzing aggregate data on 296 parliamentary democracies in 31 European countries between 1970 and 2016, the authors show that there is neither a direct nor an indirect effect of populism on voter turnout. Furthermore, the authors show that economic inequality exacerbates participatory inequality since right-wing populist parties mobilize in particular those “poor” citizens who would usually abstain from voting.

The fourth article, by *Marianne Kneuer and Mario Datts*, provides a conceptual framework for grasping the impacts of e‑democracy from a spatial perspective as well as an array of examples substantiating the framework. E‑democracy tools have been applied at all political levels, from the local to the international. Yet little is known about the differences between these levels. The authors therefore ask whether the effects of e‑democracy tools differ according to spatial context (local, national, international). Specifically, they are interested in the effect of e‑democracy on political input factors (such as involvement and mobilization of citizens) and output factors (such as responsive policies reflecting citizens’ preferences). The article offers a novel model for grasping these impacts within the respective spatial context.

The fifth contribution, by *Anja Jetschke and Sören Münch*, analyzes the impact of globalization on the “democratic design” of transnational organizations. The authors ask whether states design regional courts and parliaments as a means to exert democratic control over the executive, based on data on 76 regional organizations over a period from 1945 to 2016. Contrary to the expectations, there is no significant correlation between the level of democracy and the existence of regional institutions. Regional courts and parliaments seem to mainly serve other purposes, such as trade and conflict-related functions.

The sixth contribution, by *Daniel Kübler and Su Yun Woo*, discusses the concept and the reality of “democratic innovation” in democratic as well as in nondemocratic systems (such as China). It reviews normative assumptions and definitions prevailing in the research community and examines when and under which conditions innovations serve democratic—or other—purposes. The authors aim to identify the “democratic” as well as the “functional” dimensions of such innovations within different contexts. The contribution provides nuanced and fascinating evidence for reconsidering and rethinking “democratic innovations.”

In the seventh article, *Rainer Bauböck* initiates a theoretical debate on democratic norms and immigration control in the European Union. He claims that the immigration control powers of democratic states exist not because these states are democratic but because they are independent states. Second, he argues that democratic norms provide clear, positive reasons for promoting free international movement and admission claims for migrants and refugees. Third, he interprets the current disputes over immigration policy in the EU as a result of a conflict between “open“ and “closed” concepts of democracy and suggests that the concept of closure will put the future of democracy at risk.
